# circTUBD1‐hnRNPK Regulates the Proliferation and Migration of LSCC by Targeting CCAR1

**DOI:** 10.1002/cam4.70834

**Published:** 2025-03-25

**Authors:** Yufeng Xu, Shijie Qiu, Zhisen Shen, Jingjing Chen

**Affiliations:** ^1^ Department of Otorhinolaryngology‐Head and Neck Surgery The Affiliated LiHuiLi Hospital of Ningbo University Ningbo China

**Keywords:** CCAR1, circRNA, circTUB1, hnRNPK, LSCC, tumor proliferation and migration, tumorigenesis

## Abstract

**Background:**

Laryngeal squamous cell carcinoma (LSCC) is one of the most prevalent malignancies of the head and neck region. Circular RNAs (circRNAs) have been found to exhibit abnormal expression patterns in various tumors and play pivotal roles in tumorigenesis and tumor progression.

**Methods:**

Functional assays assessed proliferation, migration, and invasion. Mechanistic studies were performed to explore the interaction between circTUBD1 and heterogeneous nuclear ribonucleoprotein K (hnRNPK), as well as its regulation of Cell Cycle and Apoptosis Regulator 1 (CCAR1). In vivo experiments confirmed circTUBD1's role in tumor growth and metastasis.

**Results:**

We discovered that circTUBD1 is significantly upregulated in LSCC and promotes the proliferation, invasion, and migration of LSCC cells. circTUBD1 forms a circRNA‐protein complex with hnRNPK and facilitates LSCC progression by regulating CCAR1. Furthermore, in vivo experiments in mice demonstrated that silencing circTUBD1 inhibits the proliferation and metastasis of LSCC.

**Conclusions:**

This study provides evidence that circTUBD1 is a potential tumor marker for LSCC and underscores the therapeutic potential of targeting circTUBD1 in this cancer type.

## 1 Introduction

Laryngeal carcinoma is a common malignancy of the head and neck region [1], accounting for approximately 5.7%–7.6% of all malignancies, with 40% of patients diagnosed at stage III or IV [2]. Over 90% of laryngeal cancers are pathologically classified as laryngeal squamous cell carcinoma (LSCC), with a higher incidence among males than females [3]. Despite advancements in the treatment of LSCC in recent years, the overall cure rate and survival rate of patients remain low [4]. Therefore, unraveling the molecular mechanisms underlying LSCC development is crucial for developing effective therapies.

Circular RNAs (circRNAs) are a class of single‐stranded, closed loop RNA molecules that play vital roles in biogenesis, regulation, localization, degradation, and modification. When acting as miRNA sponges, circRNAs can regulate the transcription of target genes [5, 6]. Furthermore, they can interact with proteins to participate in regulating protein stability or other biological functions [7, 8, 9, 10]. Consequently, circRNAs are involved in the regulation of various physiological or pathological processes, such as epithelial‐mesenchymal transition [11], cardiovascular and cerebrovascular diseases [12, 13], autoimmune diseases [14], and tumorigenesis and development [15, 16, 17]. In cancer, circRNAs participate in tumorigenesis and development through multiple mechanisms, including influencing cell cycle progression [18], tumorigenesis [19], tumor proliferation, tumor migration [20], apoptosis [21], and angiogenesis [22]. Due to their stability, circRNAs are often studied as diagnostic biomarkers or therapeutic targets [15, 23, 24]. Therefore, exploring the specific mechanisms of circRNA involvement in LSCC is crucial for discovering potential therapeutic approaches for managing this malignancy.

For instance, in hepatocellular carcinoma (HCC), Cell Cycle and Apoptosis Regulator 1 (CCAR1) is overexpressed and associated with poor prognosis [25]. Moreover, circRNAs have been shown to negatively regulate cancer stem cells in HCC by physically binding to the CCAR1 complex through FMRP [26]. Similarly, hsa_circ_0038646 promotes proliferation and invasion by colorectal cancer cells through the miR‐331‐3p/GRIK3 axis [27]. However, the mechanisms underlying CCAR1's involvement in LSCC remain unclear and warrant further investigation.

Heterogeneous nuclear ribonucleoprotein K (hnRNPK) is a nucleic acid‐binding protein that serves as a docking platform for integrating transduction pathways into nucleic acid‐directed processes [28]. Recently, this protein has emerged as a significant player in oncogenic processes, playing a crucial role in tumorigenesis, tumor proliferation, and tumor migration [29]. However, the underlying mechanisms by which hnRNPK exerts these effects remain unclear.

Here we analyzed the expression profile of circRNAs in LSCC and identified a circRNA derived from the TUBD1 gene (hsa_circ_0044894) designated circTUBD1. Compared to matched adjacent non‐tumor tissues, circTUBD1 was upregulated in LSCC. Mechanistically, circTUBD1 can form a circRNA‐protein complex with hnRNPK and promote the proliferation and migration of LSCC by regulating CCAR1. Our results suggest that circTUBD1 plays a significant role in LSCC and has the potential to serve as a novel tumor marker for LSCC.

## 2 Materials and Methods

### 2.1 Clinical Specimens, Experimental Animals, and Cell Culture

Tissue specimens from 40 patients with LSCC and their respective controls were collected from the Affiliated LiHuiLi Hospital of Ningbo University. Participants provided written informed consent, and the research protocol was approved by the Medical Ethics Committee of the Affiliated LiHuiLi Hospital of Ningbo University. Fresh tumor tissues were rapidly frozen in liquid nitrogen for subsequent RNA extraction or fixed in paraformaldehyde for subsequent immunohistochemical analysis. C57BL/6 mice (6–8 weeks old) were purchased from Zhejiang Weitong Lihua Experimental Animal Technology Company and maintained under specific pathogen‐free conditions. Immunohistochemical experiments were conducted by observing tumor formation after tumor transplantation, measuring the tumor volume, and recording the time to tumor formation.

The human bronchial epithelial cell (HBEC), AMC‐HN‐8, TU212, and Hep‐2 cell lines were purchased from Sebastian Biotechnology Company and were cultured in DMEM medium containing 10% fetal bovine serum (FBS) at 37°C in a 5% CO_2_ incubator.

### 2.2 qRT‐PCR

Total RNA was isolated from 3 × 10^6^ cells using Trizol reagent (Ambion, USA) according to the manufacturer's instructions. Subsequently, the RNA (500 ng) was reverse‐transcribed into cDNA using HiScript II Q Select RT SuperMix (Vazyme, China), also following the manufacturer's protocol. qRT‐PCR analysis was performed on a fluorescence‐based quantitative PCR instrument (Roche, Switzerland) using SYBR Green mix (Vazyme, China). Each reaction was run in triplicate to ensure reproducibility and accuracy. The primers used in the experiment are listed in Table S1.

### 2.3 RNase R Treatment and Sanger Sequencing

Total RNA (1 μg) was treated with 3 U of RNase R (Beyotime, China) at 37°C for 15 min to enrich circular RNAs. Primers specific for circTUBD1 and other genes with the following sequences were obtained from Tsingke: For: TCTCTCAACAAGGACCTGCAT; Rev: ATGAAGAAGGAGGGCGTCTG. The PCR products of the amplified circTUBD1 were separated on a 1% agarose gel. Subsequently, Sanger sequencing was performed to confirm the back‐splice junction site of circTUBD1, thereby validating its circular structure.

### 2.4 RNA FISH

The experimental cells were counted and resuspended at a density of 100,000 cells/mL. A droplet of the cell suspension was then placed onto a glass slide, followed by fixation with 4% paraformaldehyde for 20 min. Subsequently, they were digested with 500 μL of Proteinase K (20 μg/mL). Following digestion, hybridization was performed overnight using a circTUBD1‐specific probe at a concentration of 125 nM. After hybridization, slides were incubated with (DIG)‐HRP antibody, and the nuclei were counterstained with 500 μL of DAPI (5 μg/mL). Images were captured using a fluorescence microscope.

### 2.5 CCK‐8 and EdU Assays

Based on the instructions provided by manufacturer NCM, we evaluated the proliferation of LSCC cell lines using the CCK‐8 Cell Proliferation Kit. Cells were seeded at a density of 3000 cells per well in 96‐well plates and cultured for 0, 24, 48, 72, and 96 h, respectively. Subsequently, 10 μL of CCK‐8 solution was added to each well and incubated for 1 h. The absorbance values at 450 nm were measured using a microplate reader.

For the EdU assay, EdU was diluted to 20 μM in complete medium, and 100 μL of the diluted EdU working solution was added to each well of a 96‐well plate, resulting in a final concentration of 10 μM. Cells were further incubated at 37°C for 2 h. Following fixation, cells were stained according to the manufacturer's protocol (Beyotime, China) for 35 min in the dark. The cells were then observed under a confocal microscope.

### 2.6 Transwell Migration Assay

Transwell filters (Corning, USA) were pre‐coated with or without 8 μg/μL of Matrigel on the bottom side, were placed in 24‐well plates containing DMEM medium. Subsequently, 40,000 AMC‐HN‐8 or TU212 cells in 200 μL were added to the upper chamber of the Transwell system and incubated at 37°C for 24 h to allow for migration. Migrated cells were fixed with 4% Paraformaldehyde Fix Solution for 0.5 h, followed by staining with crystal violet for 10 min.

### 2.7 RNA Pull‐Down Assay

Biotinylated circTUBD1 antisense probes and sense probes were incubated for 2 h to produce probe‐coated magnetic beads. A total of 1.5 million AMC‐HN‐8 or TU212 cells were collected. After lysis and washing, the lysates of AMC‐HN‐8 or TU212 cells were incubated with the magnetic beads to form circTUBD1‐protein complexes. After elution of the complexes, circTUBD1‐protein interactions were detected by Western blot or mass spectrometry.

### 2.8 Crosslinking RIP Assay

The Magna RIP RNA‐Binding Protein Immunoprecipitation Kit (Beyotime, China) was used to quantitatively assess protein expression following the manufacturer's instructions. 1.5 million AMC‐HN‐8 or TU212 cells were subjected to crosslinking to stabilize RNA‐protein interactions. The cells were then incubated with magnetic beads coupled to either hnRNPK‐specific antibodies (Proteintech, China) or control IgG (Proteintech, China). After extensive washing, the immunoprecipitated RNA was extracted and reverse‐transcribed into cDNA. The co‐precipitated RNA was detected by qRT‐PCR, allowing for the quantification of the interaction between hnRNPK and specific RNAs, including circTUBD1.

### 2.9 Western Blot Analysis

After preparing the total protein lysates, they were heated at 95°C for 10 min and then transferred to a 15% SDS‐PAGE gel for separation, followed by transfer to a PVDF membrane. After blocking non‐specific binding, the membrane was incubated with primary antibodies overnight at 4°C, followed by incubation with secondary antibodies (Proteintech, China) for 60 min at room temperature. The bands were detected using a gel imaging system. The antibodies used in this Western blot analysis were as follows: anti‐GAPDH (Proteintech, China), anti‐E‐cadherin (Proteintech, China), anti‐N‐cadherin (Proteintech, China), anti‐Vimentin (Proteintech, China), anti‐CCAR1 (Proteintech, China), and anti‐hnRNPK (Proteintech, China). This analysis allows for the quantification of protein expression levels and the assessment of changes in protein abundance in response to various experimental conditions.

### 2.10 Statistical Analysis

All experiments in this study were repeated more than three times, and all data were expressed as mean ± SD. SPSS 20.0 and GraphPad Prism7 were used for data analysis. Chi‐square tests were used to analyze clinical features, and *t*‐tests were used to compare the differences between the two groups. Statistical significance was set at *p* < 0.05.

## 3 Results

### 3.1 circTUBD1 is Upregulated in LSCC

**FIGURE 1:**
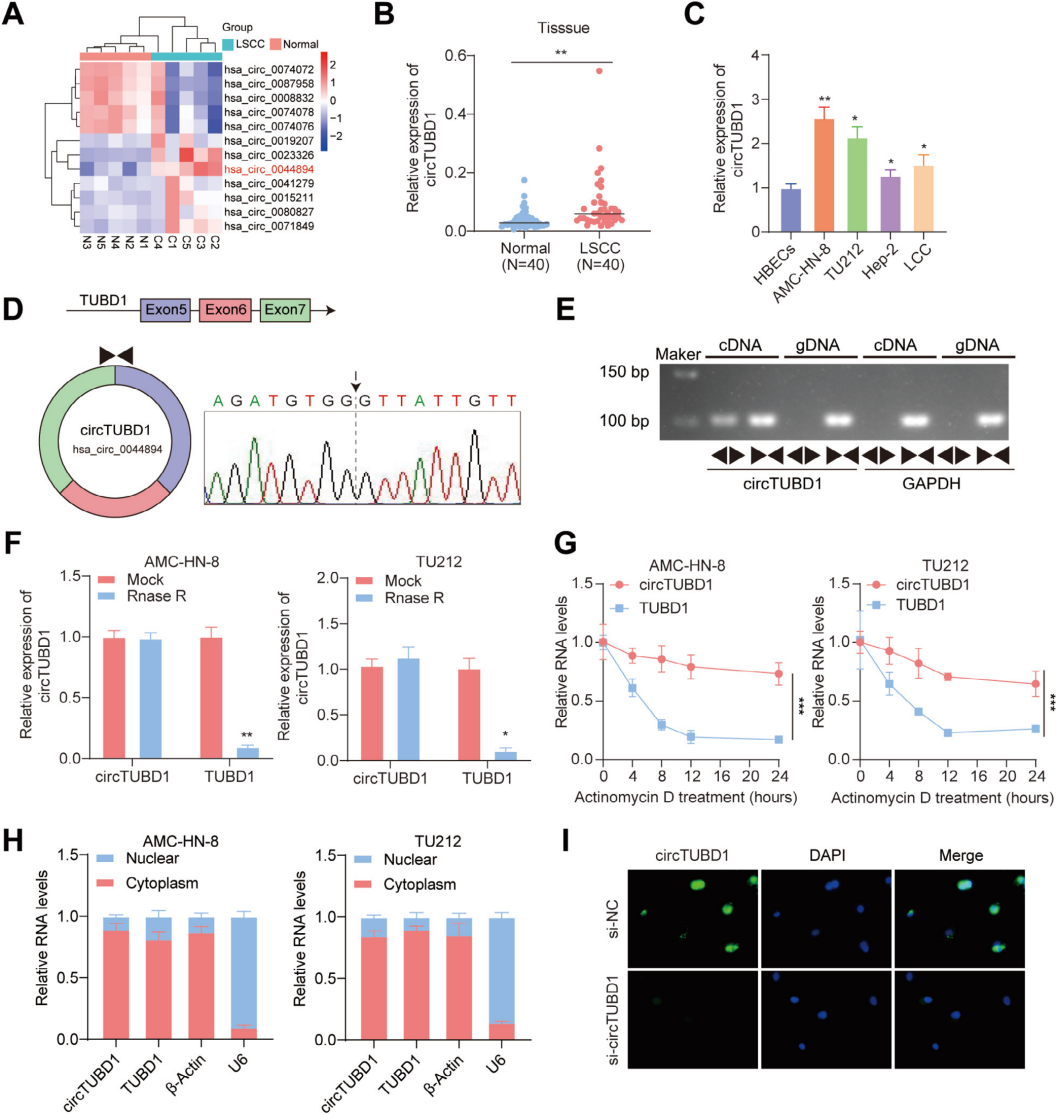
circTUBD1 is Upregulated in LSCC. (A) A heatmap depicts the most differentially expressed circRNAs between LSCC tissue samples and non‐tumor tissue samples, with the top six upregulated or downregulated genes selected. (B) The expression level of circTUBD1 in 40 pairs of LSCC tissues and their corresponding normal tissue samples was quantified using qRT‐PCR (*p* < 0.01). (C) The levels of circTUBD1 in HBECs, AMC‐HN‐8, TU212, Hep‐2, and LCC cells were determined by qRT‐PCR. (D) The reverse splicing junction site of circTUBD1 was confirmed by Sanger sequencing. (E) Gel electrophoresis was performed to analyze the PCR products of circTUBD1, linear TUBD1, and GAPDH. (F) After RNase R and Mock treatments, qRT‐PCR was used to detect the expression levels of circTUBD1 and linear TUBD1. (G) After treatment with actinomycin D for different durations, the expression levels of circTUBD1 and linear TUBD1 were detected using qRT‐PCR. (H) qRT‐PCR was employed to measure the expression levels of circTUBD1 in AMC‐HN‐8 and TU212 cells, where RNA was isolated from the cytoplasmic and nuclear fractions. (I) FISH staining confirmed the cytoplasmic localization of circTUBD1. Scale bar, 100 μm. *p < 0.05, **p < 0.01.

Initially, we screened five pairs of LSCC tissues using the Arraystar circRNA microarray. A clustered heatmap analysis focusing on the top 12 dysregulated circRNA expressions revealed a significant upregulation of hsa_circ_0044894 (Figure 1A). To further validate the abnormal expression of hsa_circ_004489 (designated as circTUBD1 in subsequent text for clarity) in LSCC tissues, we employed qPCR to assess the expression levels of circTUBD1 in 40 pairs of LSCC tissues and their corresponding normal tissue samples. The results demonstrated that circTUBD1 was significantly upregulated in LSCC tissues compared to normal tissues (Figure 1B), consistent with the findings from the RNA sequencing analysis. Compared to HBECs, similar results were confirmed in four LSCC cell lines, including AMC‐HN‐8, TU212, Hep‐2, and LCC (Figure 1C). In this study, we selected AMC‐HN‐8 and TU212, which exhibited higher expression levels, as the subsequent experimental cell lines for further investigation.

Subsequently, we designed distinct primers to characterize the circRNA through qRT‐PCR. Sanger sequencing of the amplification products revealed the presence of a back‐splice junction connecting exons 5–7 of TUBD1 (Figure 1D), thereby identifying the circRNA derived from TUBD1, designated as circTUBD1 (hsa_circ_0044894). To validate its authenticity as a circRNA, we employed an additional divergent primer (spanning the circularization site) alongside convergent primers to test the head‐to‐tail splicing of endogenous circTUBD1. Notably, circTUBD1 could be amplified by different primers from cDNA but not from genomic DNA (gDNA) (Figure 1E), indicating its circular nature. In experiments using RNase R and actinomycin D to assess the stability of circTUBD1, we found that RNase R degraded linear transcripts but spared the circular transcripts (Figure 1F), demonstrating the resistance of circTUBD1 to RNase R digestion. Furthermore, upon actinomycin D treatment, circTUBD1 exhibited greater stability compared to its linear counterpart (Figure 1G). These results collectively suggest that circTUBD1 is a bona fide circRNA with the expected molecular structure and biochemical properties, present in AMC‐HN‐8 and TU212 cells. Additionally, qPCR revealed that circTUBD1 was primarily localized within the cytoplasm of AMC‐HN‐8 and TU212 cells (Figure 1H). This subcellular localization was further confirmed by RNA‐FISH analysis, which also showed a predominant distribution of circTUBD1 within the cytoplasm (Figure 1I).

### 3.2 circTUBD1 Promotes LSCC Cell Proliferation and Migration

**FIGURE 2:**
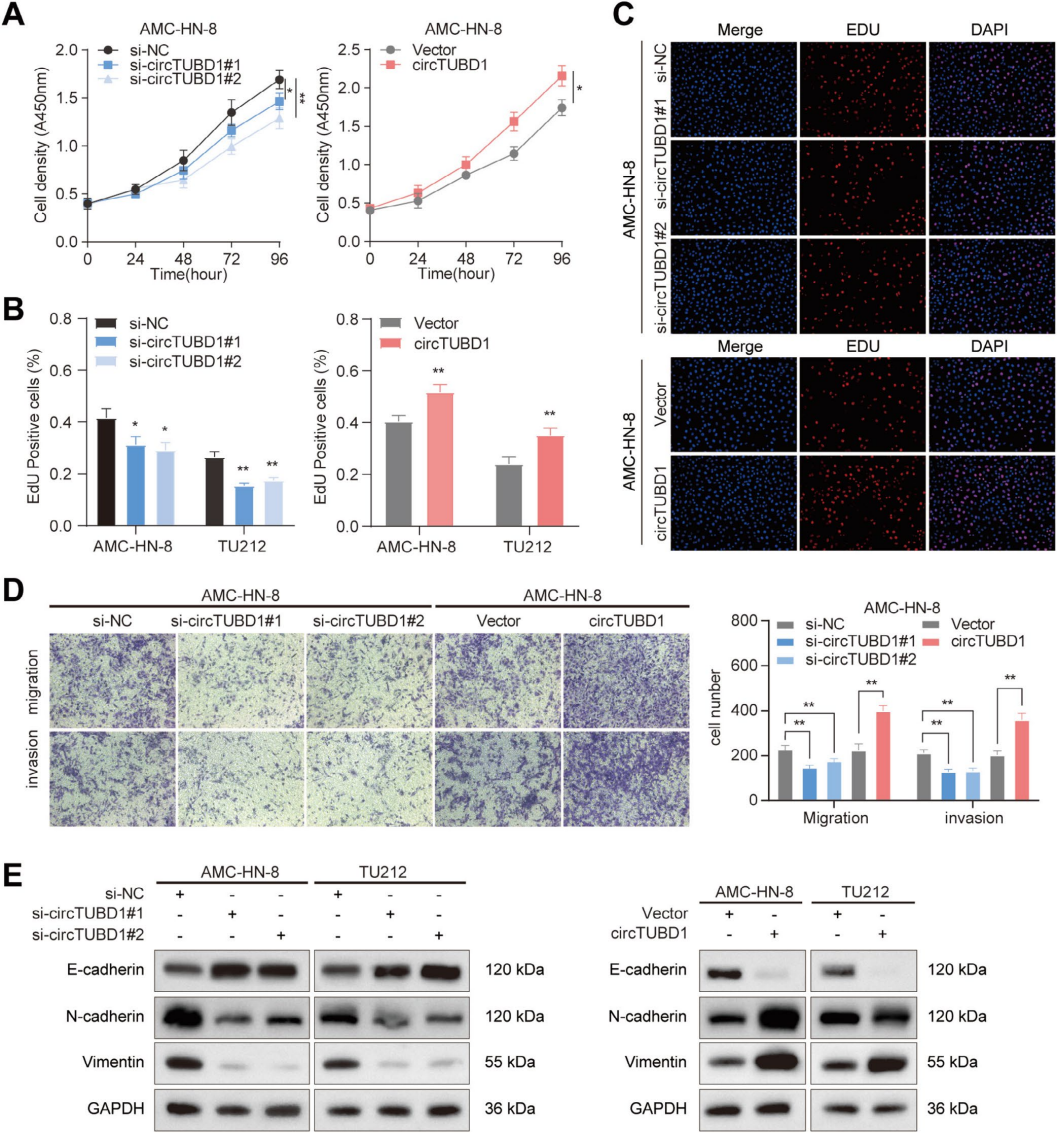
Alterations in LSCC Progression Following Modulation of circTUBD1 Levels. (A–C) The proliferation capacity of AMC‐HN‐8 cells was assessed after altering circTUBD1 levels using CCK‐8 and EDU assays. (D) The migration and invasion capabilities of AMC‐HN‐8 cells were evaluated through Transwell assays following modulation of circTUBD1 levels. (E) Western blot analysis was performed to detect the protein expression levels of E‐cadherin, N‐cadherin, and Vimentin in AMC‐HN‐8 cells after altering circTUBD1 levels. *p < 0.05, **p < 0.01.

To investigate the functional role of circTUBD1 in LSCC, we used small interfering RNA (siRNA) to silence circTUBD1 and constructed plasmids to overexpress circTUBD1. qRT‐PCR assays confirmed efficient transfection in AMC‐HN‐8 and TU212 cells after 48 h (Figure S1A). Subsequently, circTUBD1#1 and si‐circTUBD1#2, which exhibited better silencing efficiency, were selected for CCK‐8 and EDU assays. The results demonstrated that overexpression of circTUBD1 promoted proliferation in AMC‐HN‐8 and TU212 cells, while silencing of circTUBD1 inhibited their proliferative capacity (Figures 2A–C and Figure S1B,C). To explore the impact of circTUBD1 on LSCC cell invasion and migration, Transwell assays were performed. The findings revealed that silencing circTUBD1 significantly inhibited migration and invasion by AMC‐HN‐8 cells, whereas overexpression of circTUBD1 markedly enhanced these processes (Figure 2D). Consistent results were also observed in TU212 cells (Figure S1D). Epithelial‐mesenchymal transition (EMT) is a pivotal process in cancer cell metastasis [30], and circRNAs play specific biological roles in EMT [7]. Western blot analysis showed that silencing circTUBD1 led to increased E‐cadherin expression and decreased N‐cadherin and vimentin expression, with the opposite effects observed upon circTUBD1 overexpression (Figure 2E). In summary, circTUBD1 promotes LSCC progression in terms of proliferation, invasion, and migration. However, the underlying molecular mechanisms warrant further investigation.

### 3.3 circTUBD1 Promotes the Proliferation and Migration of LSCC by Regulating CCAR1

**FIGURE 3:**
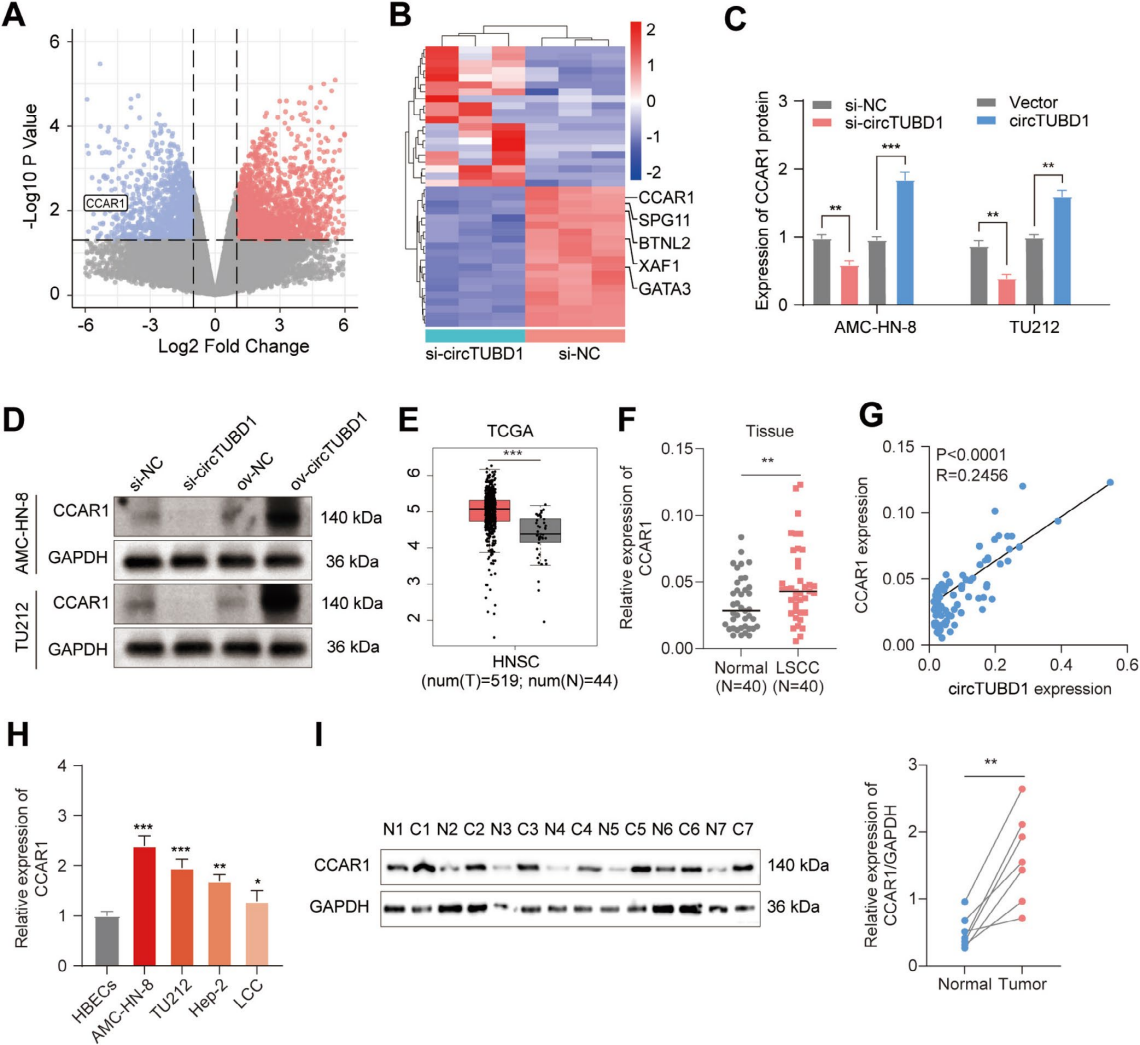
Regulation of CCAR1 by circTUBD1. (A, B) Volcano plots and clustering heatmaps based on RNA‐Seq transcriptomic data from AMC‐HN‐8 cells were generated after silencing circTUBD1 or its control. (C) The expression of CCAR1 in AMC‐HN‐8 and TU212 cells was detected using qRT‐PCR after altering the levels of circTUBD1. (D) Western blot analysis was performed to assess CCAR1 protein expression following alterations in circTUBD1 levels. (E) CCAR1 expression in LSCC tissues was examined using data from the TCGA database. (F) qRT‐PCR was utilized to quantify CCAR1 levels in 40 paired LSCC samples. (G) Correlation analysis was conducted between circTUBD1 and CCAR1 expression in 40 LSCC tissues. (H) qRT‐PCR was performed to detect CCAR1 levels in HBECs, AMC‐HN‐8, TU212, Hep‐2, and LCC cell lines. (I) Western blot analysis was used to detect CCAR1 protein levels in 7 paired LSCC samples. *p < 0.05, **p < 0.01, ***p < 0.001.

To elucidate the molecular mechanism underlying the role of circTUBD1 in promoting LSCC progression, we first conducted RNA‐Seq transcriptome profiling to compare gene expression between circTUBD1‐silenced and control conditions. The volcano plot analysis revealed downregulation of CCAR1 among the identified genes (Figure 3A), while the heatmap further highlighted the downregulation of CCAR1, along with SPG11, BTNL2, XAF1, and GATA3 (Figure 3B). However, qRT‐PCR validation demonstrated that only CCAR1 expression was significantly reduced upon circTUBD1 silencing and conversely upregulated upon circTUBD1 overexpression (Figure 3C). Consistent with these findings, Western blot analysis in AMC‐HN‐8 and TU212 cells confirmed the regulation of CCAR1 protein levels by circTUBD1 (Figure 3D).

To validate whether CCAR1 is a potential regulatory target of circTUBD1 in LSCC in clinical settings, we utilized the TCGA database [31]. Analysis of TCGA data revealed upregulated CCAR1 expression in LSCC tissues (Figure 3E), which was corroborated by qRT‐PCR results from 40 paired LSCC samples (Figure 3F) and further supported by Western blot analysis (Figure 3I). Notably, a positive correlation was observed between circTUBD1 and CCAR1 expression levels in the 40 LSCC clinical tissue specimens (Figure 3G). Additionally, compared to HBECs, upregulated CCAR1 expression was confirmed in four LSCC cell lines, including AMC‐HN‐8, TU212, Hep‐2, and other LSCC cell lines (Figure 3H).

**FIGURE 4:**
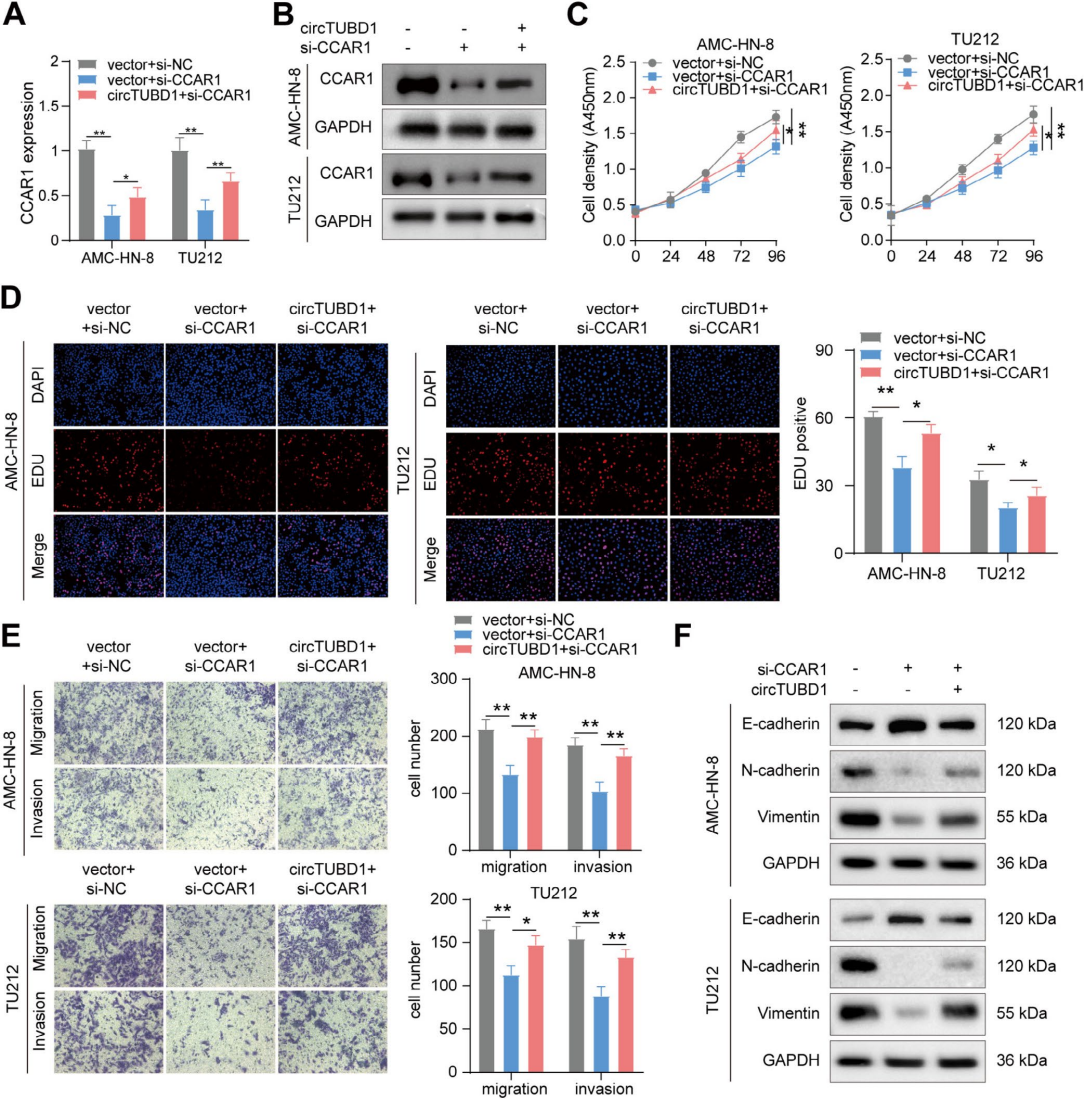
circTUBD1 Promotes Proliferation and Migration of LSCC by Regulating CCAR1. (A, B) Western blot and qPCR were used to validate the expression levels of CCAR1. (C, D) Cell proliferation was assessed using CCK‐8 and EDU assays. (E) The migration and invasion capabilities of cells were evaluated through Transwell assays. (F) The protein expression levels of E‐cadherin, N‐cadherin, and Vimentin in AMC‐HN‐8 and TU212 cells were assessed through Western blot analysis. *p < 0.05, **p < 0.01.

To investigate the potential biological functions of CCAR1 in LSCC cells, CCAR1 was first silenced using small interfering RNA (siRNA). qPCR and Western blot assays confirmed the effective silencing of CCAR1 in AMC‐HN‐8 and TU212 cells, which was subsequently upregulated by circTUBD1 (Figure 4A,B). Functional rescue experiments were then conducted to explore whether circTUBD1 drives LSCC progression through CCAR1 mediation. CCK‐8 and EDU assays revealed that, compared to the control, CCAR1 silencing reduced the proliferation of AMC‐HN‐8 and TU212 cells, whereas circTUBD1 overexpression rescued this effect and promoted cell proliferation (Figure 4C,D). Transwell assays demonstrated that CCAR1 silencing significantly inhibited the migration and invasion of AMC‐HN‐8 and TU212 cells, while circTUBD1 overexpression significantly promoted migration and invasion in CCAR1‐silenced cells (Figure 4E). Additionally, the CCK‐8 and EDU experiments indicated that silencing of circTUBD1 decreased the proliferation of AMC‐HN‐8 and TU212 cells, while overexpression of CCAR1 rescued this effect and promoted cell proliferation (Figure S2A,B). The Transwell assays similarly demonstrated that silencing of circTUBD1 reduced the migration and invasion of AMC‐HN‐8 and TU212 cells, and that overexpression of CCAR1 rescued this effect, promoting the migration and invasion of the cells (Figure S2C). In addition, Western blot analysis showed that silencing CCAR1 led to increased expression of E‐cadherin, decreased expression of N‐cadherin and vimentin, while overexpression of circTUBD1 rescued this effect (Figure 4F). In conclusion, circTUBD1 promotes the proliferation and metastasis of LSCC by regulating CCAR1.

### 3.4 circTUBD1 Interacts With hnRNPK in LSCC Cells

**FIGURE 5:**
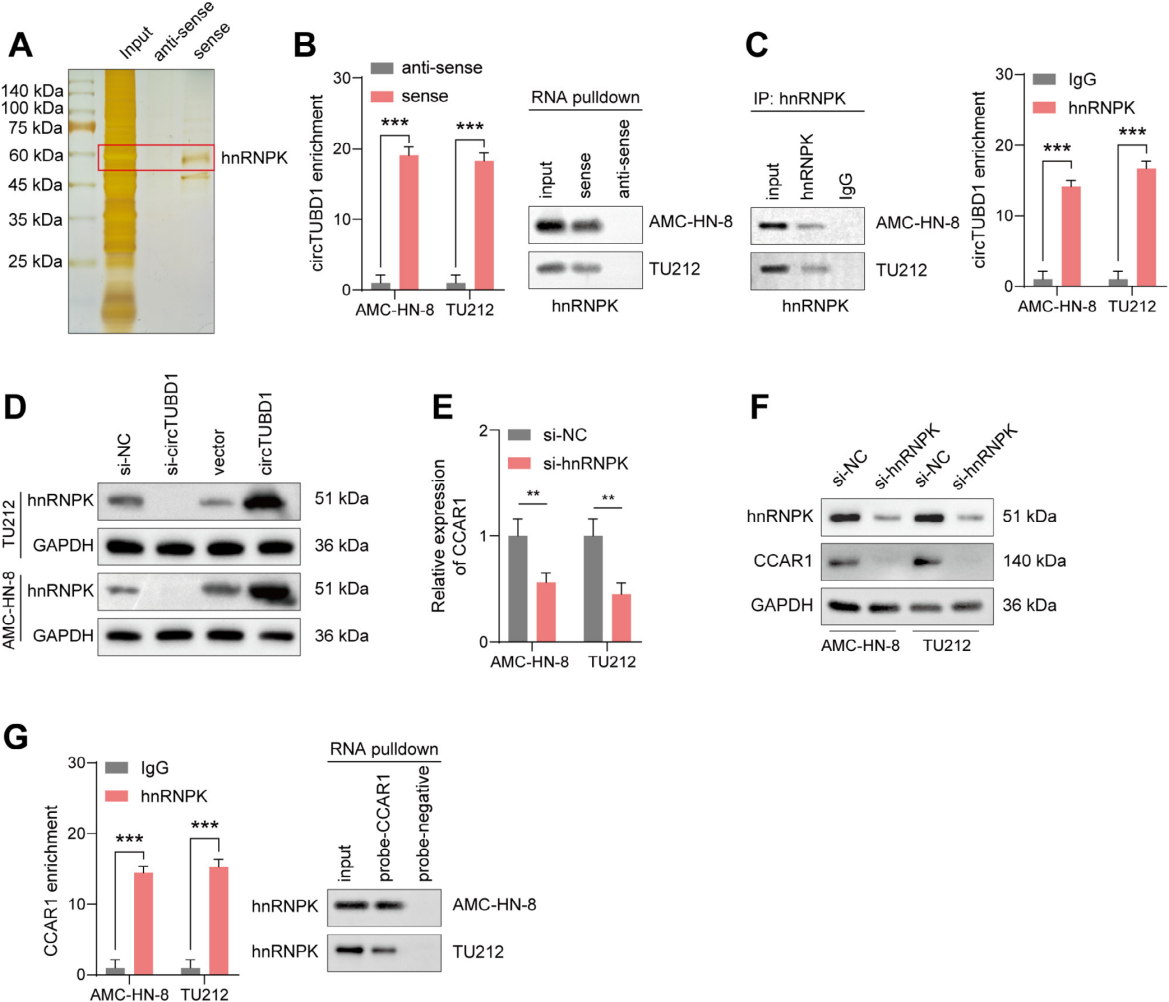
circTUBD1 Interacts with hnRNPK in LSCC Cells. (A) Silver staining were employed to identify proteins that bind to biotinylated circTUBD1. (B) RNA pull‐down assays were performed to detect the interaction between circTUBD1 and hnRNPK in cells. (C) RIP assays were conducted to verify the interaction between endogenous hnRNPK and circTUBD1 in cells. (D) After overexpressing or knocking down circTUBD1, Western blot analysis was used to detect the expression of hnRNPK. (E, F) Following hnRNPK knockdown, qPCR and WB were performed to assess the expression of CCAR1. (G) RNA pull‐down assays were performed to detect the interaction between CCAR1 and hnRNPK in cells. **p < 0.01, ***p < 0.001.

To further elucidate the molecular mechanism by which circTUBD1 regulates CCAR1 in LSCC cells, we employed biotin‐labeled circTUBD1 probes and mass spectrometry analysis in RNA pull‐down assays to screen for proteins that interact with circTUBD1 (Figure 5A). Mass spectrometry data revealed that hnRNPK was the top‐scoring circTUBD1‐binding protein based on its score or unique peptide count. RNA pull‐down experiments confirmed that the sense probe of circTUBD1 could effectively and specifically enrich hnRNPK (Figure 5B). Additionally, RIP assays showed that circTUBD1 was enriched in the complexes precipitated by anti‐hnRNPK antibodies compared to control IgG (Figure 5C). Western blot analysis revealed that overexpression of circTUBD1 increased hnRNPK expression, while silencing circTUBD1 decreased hnRNPK expression (Figure 5D). Similarly, qPCR and Western blot assays demonstrated that silencing hnRNPK significantly reduced CCAR1 expression (Figure 5E,F). RIP assays further confirmed that CCAR1 was enriched in the complexes precipitated by anti‐hnRNPK antibodies compared to control IgG (Figure 5G). These results collectively demonstrate that circTUBD1 can bind to hnRNPK to form a circRNA‐protein complex in LSCC cells, thereby regulating CCAR1.

### 3.5 circTUBD1 Promotes the Proliferation and Migration of LSCC by Regulating hnRNPK

**FIGURE 6:**
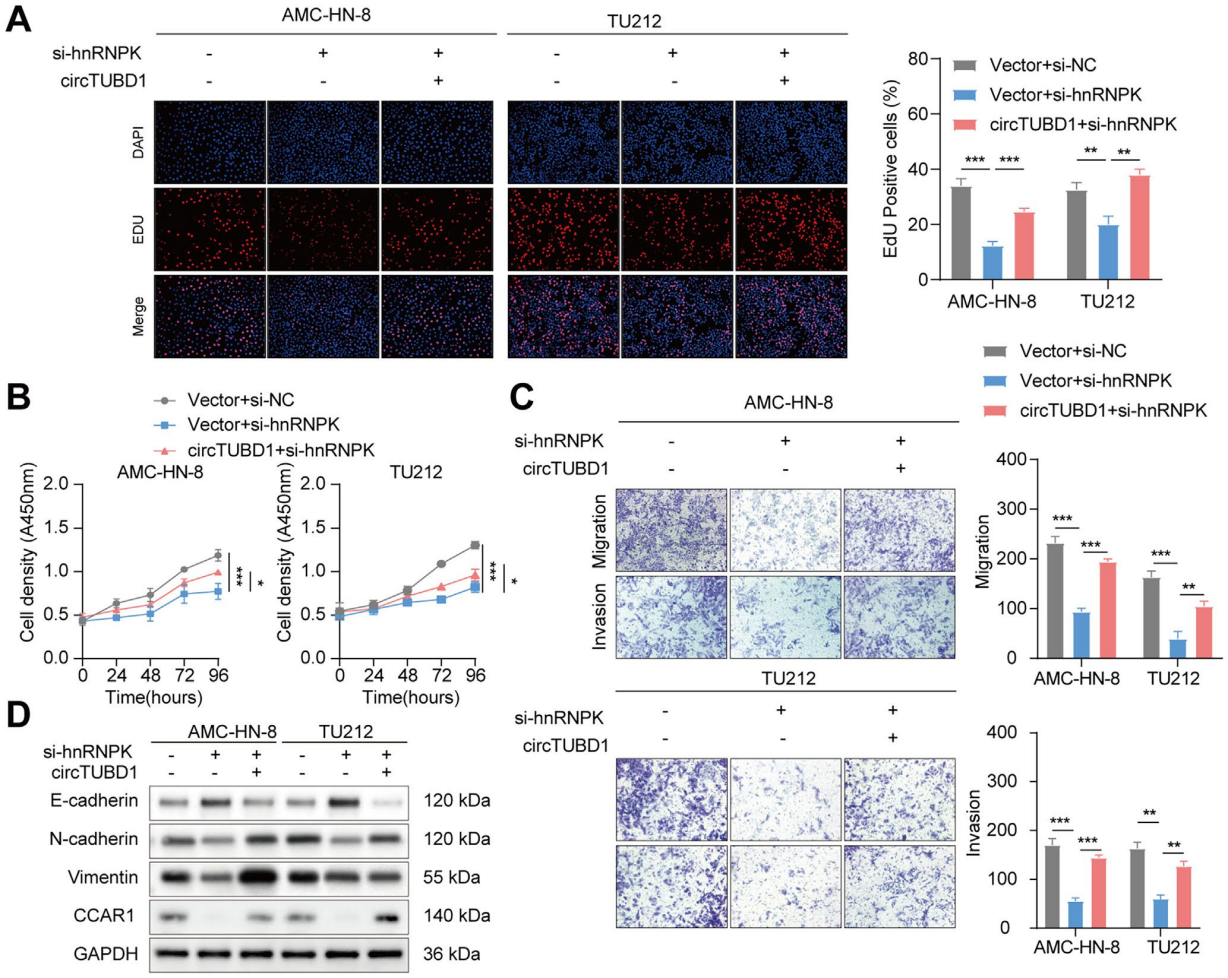
circTUBD1 Promotes Proliferation and Migration of LSCC by Regulating hnRNPK. (A, B) The proliferation capabilities of AMC‐HN‐8 and TU212 cells were evaluated using CCK‐8 and EDU assays. (C) Transwell assays were performed to assess the migration and invasion abilities of AMC‐HN‐8 and TU212 cells. (D) Western blot analysis was conducted to detect the protein expression levels of E‐cadherin, N‐cadherin, Vimentin and CCAR1. *p < 0.05, **p < 0.01, ***p < 0.001.

To investigate whether circTUBD1 drives LSCC progression through hnRNPK mediation, functional rescue experiments were performed. CCK‐8 and EDU assays showed that, compared to the control, silencing hnRNPK reduced the proliferation of AMC‐HN‐8 and TU212 cells, whereas circTUBD1 overexpression rescued this effect and promoted cell proliferation in si‐hnRNPK‐treated cells (Figure 6A,B). Additionally, Transwell assays demonstrated that silencing hnRNPK significantly inhibited the migration and invasion of AMC‐HN‐8 and TU212 cells, while circTUBD1 overexpression significantly promoted migration and invasion in si‐hnRNPK‐treated cells (Figure 6C). Western blot analysis revealed that silencing hnRNPK increased E‐cadherin expression and decreased N‐cadherin, vimentin, and CCAR1 expression, whereas circTUBD1 overexpression had the opposite effect (Figure 6D). In conclusion, circTUBD1 regulates hnRNPK to promote the proliferation and metastasis of LSCC.

### 3.6 Silencing of circTUBD1 Inhibits Proliferation and Invasion by LSCC Cells In Vivo

**FIGURE 7:**
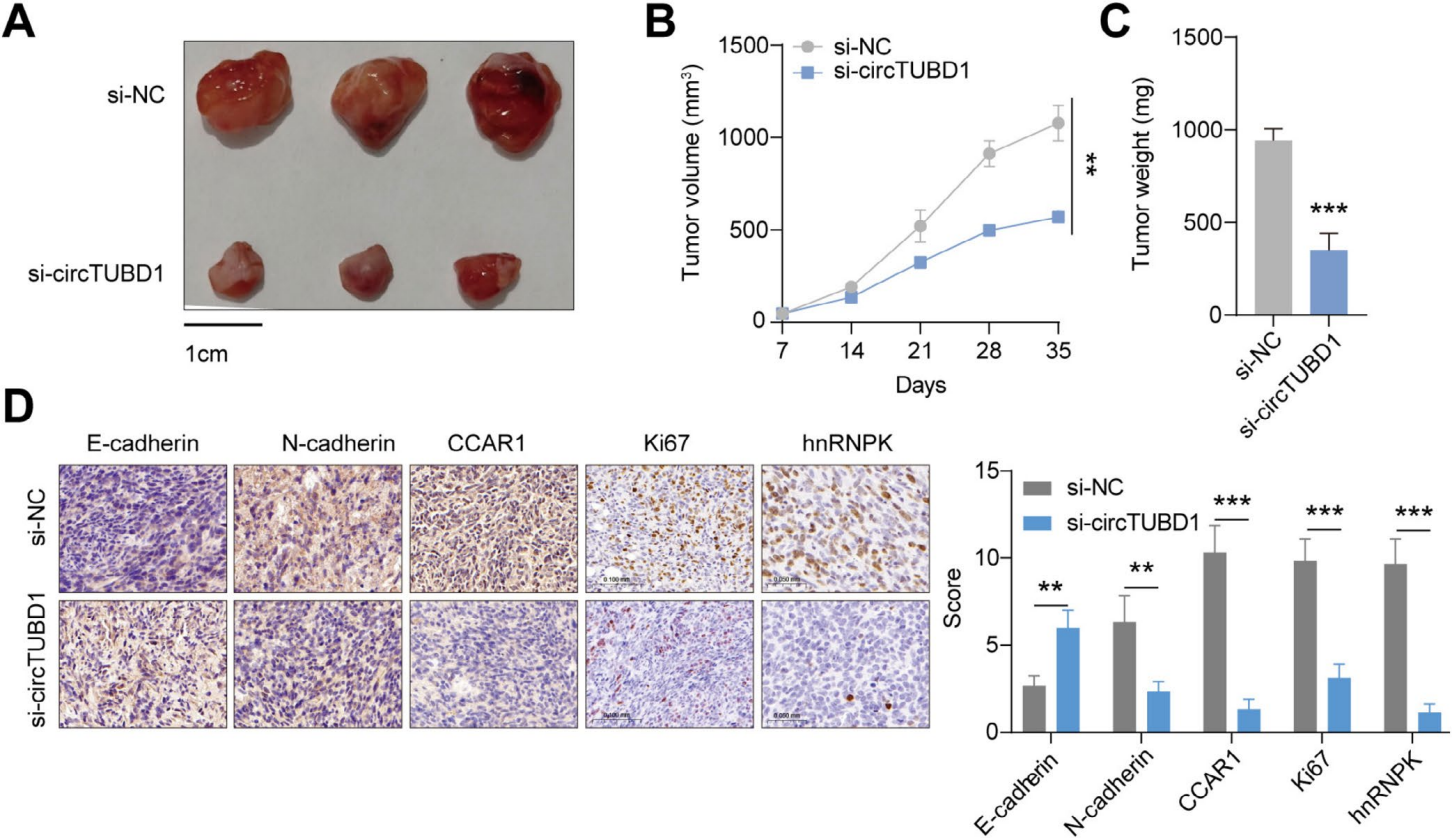
Silencing of circTUBD1 Inhibits the Proliferation and Migration of LSCC Cells in vivo. (A–C) Volume and weight of subcutaneous xenograft tumors were measured. (D) IHC staining was performed to detect the expression levels of CCAR1, E‐cadherin, Ki67, hnRNPK and N‐cadherin. **p < 0.01, ***p < 0.001.

To investigate whether circTUBD1 also has the potential to impact tumor development in vivo, we transplanted AMC‐HN‐8 cells transfected with si‐circTUBD1 or control into C57BL/6 mice via subcutaneous injection. Compared to the control group, LSCC cell proliferation began to be inhibited in the si‐circTUBD1 group after 14 days, with a significant effect achieved by 35 days (Figure 7A–C). Furthermore, immunohistochemical analysis of tumor tissues dissected from these mice revealed that, compared to the control group, the si‐circTUBD1 group exhibited decreased E‐cadherin expression and increased N‐cadherin, CCAR1, Ki67, and hnRNPK expression (Figure 7D). These results further demonstrate that silencing circTUBD1 can inhibit the proliferation and metastasis of LSCC.

## 4 Discussion

The aberrant expression of circRNAs has been observed in virtually all types of cancers [32], and their involvement in tumor cell proliferation, migration, and invasion has been well established by previous studies [33]. In this research, we delved into the role of circRNAs in LSCC, specifically focusing on an upregulated circRNA (circTUBD1) identified through RNA sequencing analysis. Our findings suggest that circTUBD1 functions as an oncogene in LSCC, driving both proliferation and metastasis of the cancer cells.

In numerous studies, circRNAs have been identified as scaffolds for protein–protein interactions. For instance, in breast cancer, circDNMT1 binds to p53 and AUF1 to promote cancer progression through the activation of autophagy [34]. Similarly, in gastric cancer, CircPTK2 interacts with the PABPC1 protein in bladder cancer (BCa) cells [35]. Our research findings provide convincing evidence supporting the role of circRNAs as scaffolds for protein interactions. In this study, circTUBD1 formed a circRNA‐protein complex with hnRNPK in LSCC cells, contributing to its pathological effects. Interestingly, we also discovered that circTUBD1 promotes the proliferation and metastasis of LSCC cells by regulating hnRNPK. However, the precise mode of interaction between circTUBD1 and hnRNPK and its functional consequences remain to be elucidated, highlighting the need for further investigation.

CCAR1 was initially identified as a perinuclear phosphoprotein that induces apoptosis in breast cancer cells during chemotherapy [36]. Prior studies have reported the involvement of CCAR1 in cell proliferation and apoptosis across various cell lines, including cancer cells [37, 38]. Our current study demonstrates that CCAR1 is upregulated in LSCC and functions as a target of circTUBD1, contributing to the proliferation and metastasis of LSCC under circTUBD1 regulation. However, due to the limitations of our research conditions, we did not further explore the underlying mechanisms of CCAR1's action. We speculate that CCAR1 may enhance cell stemness in LSCC through the Wnt/β‐catenin signaling pathway.

As research progresses, it is crucial to investigate the potential of circRNAs as therapeutic targets or vehicles for delivering immune‐modulatory molecules. Antisense oligonucleotides (ASOs) represent a promising strategy for targeting circTUBD1. ASOs are short, synthetic nucleic acid sequences that can specifically bind to target RNA molecules. For circTUBD1, specialized ASOs can be designed to target its unique back‐splice junction, which is a hallmark of circRNA formation. By binding to this region, ASOs can prevent circTUBD1 from interacting with its binding partners, such as hnRNPK, thereby disrupting its oncogenic functions. The ability to specifically target and modulate circRNA expression in cancer cells offers a precise and potentially powerful tool for immunotherapy. Additionally, combining circRNA‐based therapies with existing immunotherapies, such as immune checkpoint inhibitor therapy, may lead to synergistic effects and even more profound therapeutic outcomes. Recent research has highlighted the significant therapeutic potential of utilizing customized lipid nanoparticles (LNPs) encapsulating IL‐12 circRNA in combination with immune checkpoint inhibitors for the treatment of lung cancer, administered via intratumoral injection and intratracheal delivery [39]. This study underscores the promising role of circRNAs in cancer immunotherapy. Our findings in mouse models demonstrate that silencing circTUBD1 can effectively inhibit the proliferation and metastasis of LSCC, further emphasizing the therapeutic potential of targeting circRNAs in this cancer type. This discovery opens new avenues for exploration, suggesting that with the continued development of efficient delivery systems and a deeper understanding of the mechanisms underlying circTUBD1's role in LSCC, we may 1 day achieve a cure for this disease.

## Author Contributions

**Yufeng Xu:** writing – original draft, investigation, formal analysis. **Shijie Qiu:** project administration, funding acquisition. **Zhisen Shen:** supervision, funding acquisition. **Jingjing Chen:** conceptualization, writing – review and editing, funding acquisition.

## Ethics Statement

This study was approved and performed by the Ethics Committee of the Affiliated LiHuiLi Affiliated Hospital of Ningbo University(Registration number: 2022‐439). Participants provided written informed consent, and the research protocol was approved by the Medical Ethics Committee of the Affiliated LiHuiLi Hospital of Ningbo University.

## Conflicts of Interest

The authors declare no conflicts of interest.

## Supporting information


**Figure S1.** circTUBD1 Promotes Proliferation and Migration of LSCC. (A) qRT‐PCR was used to detect the levels of circTUBD1 in AMC‐HN‐8 cells and TU212 cells after silencing (si‐circTUBD1#1, si‐circTUBD1#2, and si‐circTUBD1#3) and overexpression. (B, C) CCK‐8 and EDU assays were performed to assess the proliferation capacity of TU212 cells following alterations in circTUBD1 levels. (D) Transwell assays were conducted to evaluate the migration and invasion abilities of TU212 cells after modifying circTUBD1 levels.
**Figure S2.** circTUBD1 Promotes Proliferation and Migration of LSCC by Regulating CCAR1. (A, B) Cell proliferation was assessed using CCK‐8 and EDU assays. (C) The migration and invasion capabilities of cells were evaluated through Transwell assays.
**Table S1.** RNA oligonucleotide sequences.
**Table S2.** Primer sequences.

## Data Availability

The data that support the findings of this study are available on request from the corresponding author. The data are not publicly available due to privacy or ethical restrictions.
