# Supplementary material for: circTUBD1‐hnRNPK Regulates the Proliferation and Migration of LSCC by Targeting CCAR1
Source: Cancer Med. 2025 Mar 25;14(6):e70834. doi: 10.1002/cam4.70834 (PMC11933862; doi:10.1002/cam4.70834)
Supplement: Supplementary file 1 — Figure S1. circTUBD1 Promotes Proliferation and Migration of LSCC. (A) qRT‐PCR was used to detect the levels of circTUBD1 in AMC‐HN‐8 cells and TU212 cells after silencing (si‐circTUBD1#1, si‐circTUBD1#2, and si‐circTUBD1#3) and overexpression. (B, C) CCK‐8 and EDU assays were performed to assess the proliferation capacity of TU212 cells following alterations in circTUBD1 levels. (D) Transwell assays were conducted to evaluate the migration and invasion abilities of TU212 cells after modifying circTUBD1 levels. Figure S2. circTUBD1 Promotes Proliferation and Migration of LSCC by Regulating CCAR1. (A, B) Cell proliferation was assessed using CCK‐8 and EDU assays. (C) The migration and invasion capabilities of cells were evaluated through Transwell assays. Table S1. RNA oligonucleotide sequences. Table S2. Primer sequences. [file CAM4-14-e70834-s001.docx]

**Table S1** RNA oligonucleotide sequences.

| **RNA oligos** | **Sequences** |
| --- | --- |
| si-NC | Sense: 5’- UUCUCCGAACGUGUCACGUTT -3’  Anti-sense: 5’- ACGUGACACGUUCGGAGAATT -3’ |
| si-circTUBD1#1 | Sense: 5’- [AGAUGUGGGUUAUUGUUCAAA](http://blast.ncbi.nlm.nih.gov/Blast.cgi?PROGRAM=blastn&PAGE_TYPE=BlastSearch&LINK_LOC=blasthome&QUERY=%3ehsa_circ_0044894-siRNA3%0AAGATGTGGGTTATTGTTCAAA&DATABASE=nr&EQ_MENU=Homo%C2%A0sapiens%C2%A0(taxid:9606))TT -3’  Anti-sense: 5’-UUUGAACAAUAACCCACAUCUTT -3’ |
| si-circTUBD1#2 | Sense: 5’- [AUGUGGGUUAUUGUUCAAAAC](http://blast.ncbi.nlm.nih.gov/Blast.cgi?PROGRAM=blastn&PAGE_TYPE=BlastSearch&LINK_LOC=blasthome&QUERY=%3ehsa_circ_0044894-siRNA10%0AATGTGGGTTATTGTTCAAAAC&DATABASE=nr&EQ_MENU=Homo%C2%A0sapiens%C2%A0(taxid:9606))TT -3’  Anti-sense: 5’- GUUUUGAACAAUAACCCACAUTT -3’ |
| si-CCAR1 | Sense: 5’- GGUCUAUAGUGUAACUAUAGUTT -3’  Anti-sense: 5’- UAUAGUUACACUAUAGACCCUTT -3’ |
| si-hnRNPK | Sense: 5’- GGUGUUAUUUGAAUGUUAAUATT -3’  Anti-sense: 5’- UUAACAUUCAAAUAACACCAUTT -3’ |

**Table S2** Primer sequences.

| Name | **Forward primer (5’ to 3’)** | **Reverse primer (5’ to 3’)** |
| --- | --- | --- |
| circTUBD1 | TCTCTCAACAAGGACCTGCAT | ATGAAGAAGGAGGGCGTCTG |
| CCAR1 | CTTCGTTGGATGGACCAGAC | GATCAATGCCTGTCAGAGCCT |
| U6 | CTCGCTTCGGCAGCACATATACT | ACGCTTCACGAATTTGCGTGTC |
| β-Actin | ACAGGCATCGTGATGGATTCT | CAGCAGTGGTGGTGAAGTTAT |
| GAPDH | TGCACCACCAACTGCTTAGC | GGCATGGACTGTGGTCATGAG |

**Figure S1**


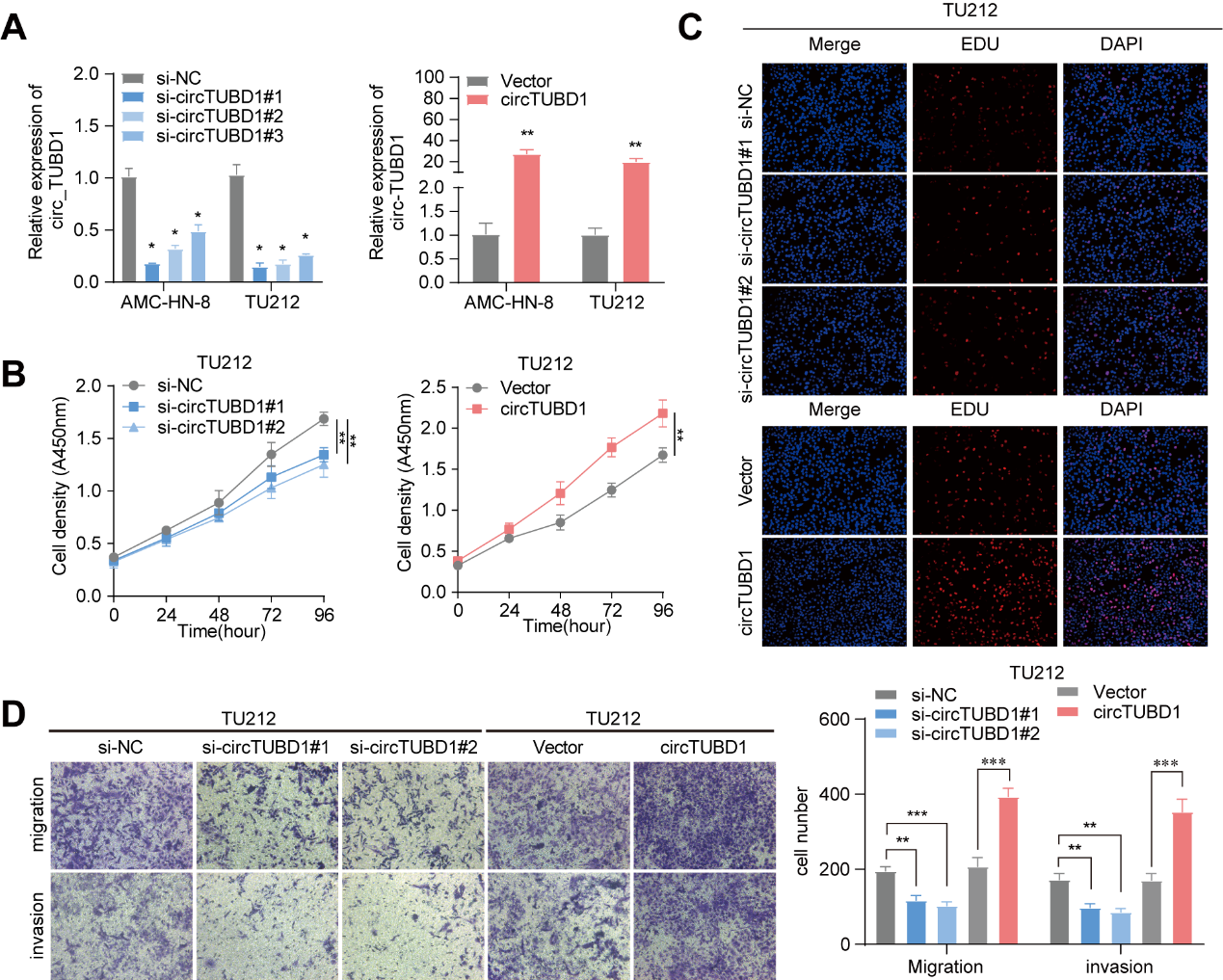


**Figure S1. circTUBD1 Promotes Proliferation and Migration of LSCC**

A. qRT-PCR was used to detect the levels of circTUBD1 in AMC-HN-8 cells and TU212 cells after silencing (si-circTUBD1#1, si-circTUBD1#2, and si-circTUBD1#3) and overexpression.

B-C. CCK-8 and EDU assays were performed to assess the proliferation capacity of TU212 cells following alterations in circTUBD1 levels.

D. Transwell assays were conducted to evaluate the migration and invasion abilities of TU212 cells after modifying circTUBD1 levels


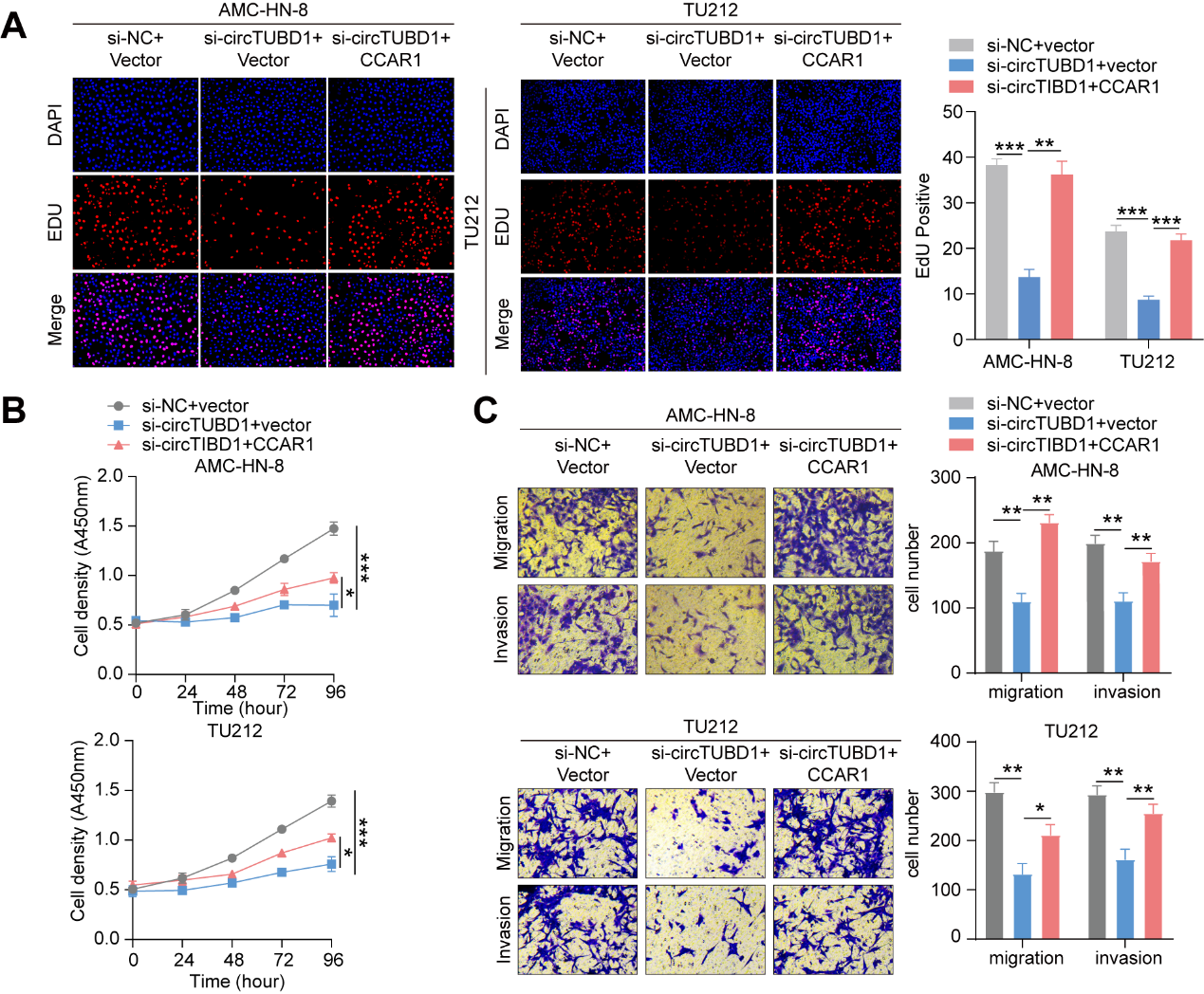


**Figure S2. circTUBD1 Promotes Proliferation and Migration of LSCC by Regulating CCAR1**

A-B. Cell proliferation was assessed using CCK-8 and EDU assays.

C. The migration and invasion capabilities of cells were evaluated through Transwell assays.
